# A Comparison of Ordered Categorical versus Discrete Choices within a Stated Preference Survey of Whole-Blood Donors

**DOI:** 10.1177/0272989X221145048

**Published:** 2022-12-24

**Authors:** Zia Sadique, John Cairns, Kaat De Corte, Sarah Willis, Alec Miners, Nick Bansback, Richard Grieve

**Affiliations:** Department of Health Services Research and Policy, London School of Hygiene and Tropical Medicine, London, UK; Department of Health Services Research and Policy, London School of Hygiene and Tropical Medicine, London, UK; Department of Health Services Research and Policy, London School of Hygiene and Tropical Medicine, London, UK; Department of Health Services Research and Policy, London School of Hygiene and Tropical Medicine, London, UK; Department of Health Services Research and Policy, London School of Hygiene and Tropical Medicine, London, UK; Health Services and Policy, University of British Columbia, Vancouver, Canada; Department of Health Services Research and Policy, London School of Hygiene and Tropical Medicine, London, UK

**Keywords:** blood donation, discrete choice experiments, stated preferences

## Abstract

There are different stated preference (SP) approaches, including discrete choice experiments (DCEs). DCEs are a popular SP approach, but in some settings, alternative ways of framing survey questions may be more appropriate. The Health Economic Modelling Of Alternative Blood Donation Strategies (HEMO) study required choice tasks to be framed so that the study could estimate the effect of attribute levels on the frequency of a behavior—in this case, blood donation. SP questions were formulated to require ordered categorical responses from a single profile of attribute levels. However, it is unknown whether this way of framing SP questions leads to estimates of marginal rates of substitution (MRS) that are different from traditional DCE choices between 2 alternative profiles. The aim of this article is to compare estimates of relative preferences from SP questions requiring ordered categorical versus discrete choice responses. We compared relative preferences elicited from the 2 approaches for a common set of attributes and levels, formulated as choice tasks for 8,933 whole-blood donors. We found that the 2 forms of survey questions provided similar MRSs estimates. For example, respondents were willing to trade off only a small increase in travel time to receive a health report, irrespective of whether the choice given was binary (DCE response; approximately 3 min) or from an ordered category (about 8 min). The finding that any differences in the estimated MRSs are not of substantive importance offers some reassurance for policy makers in that estimates of relative preference may be robust to alternative ways of framing the survey questions. These findings can encourage future studies to frame choice tasks that align with the study’s objective.

**Highlights:**

- This article compares the relative preferences from stated preference (SP) questions requiring ordered categorical versus discrete choice responses. The approaches were contrasted for blood donation service characteristics that offer opportunities to donate blood.
- The estimates of relative preferences for alternative blood donation service characteristics were similar between the 2 forms of SP approach.
- This study illustrates how SP survey questions can be formulated to provide responses on an ordered categorical scale and to estimate marginal rates of substitution between different attributes, which can be compared with those derived from discrete choice experiment (DCE) choices.
- The article highlights the potential value of considering alternative choice framings rather than relying solely on DCEs.

Stated preference (SP) studies are used to value nonmarket commodities and predict the effect of future policy changes.^1–3^ In health economics, there has been a rapid uptake of discrete choice experiments (DCEs), which are a particular form of SP design.^4^ In DCEs, respondents are required to state choices between 2 or more discrete alternatives in which at least 1 attribute level of each alternative is systematically varied across choice sets to provide the information required to infer the preference parameters of an indirect utility function.^5^ While the popularity of DCEs in health continues to increase, they may not be the most appropriate SP method in all settings. Selecting an SP approach requires consideration of the study’s objective and how that can be met by choosing an approach to framing the valuation task that responders find both realistic and understandable.^5^

An example of a setting in which an alternative SP approach may be more appropriate than a DCE is when the study is required to estimate the effect of attribute levels on the intended frequency of a behavior. The context considered in this article is that of whole-blood donation. The objective was to estimate how frequently donors would donate according to attributes and levels defined to reflect alternative future changes to the blood donation service.^6^ In this context, the frequency of donation could be included as its own attribute within a DCE. However, initial discussions with potential responders, existing blood donors, suggested that as blood donation is voluntary, framing blood donation frequency as an attribute would lead to unrealistic choices, resulting in poor engagement with the task and a high risk of misleading responses. Instead, the study developed SP choice tasks, which took the form of direct (matching) questions using the “payment card” approach, with the responder required to select their preferred “donation frequency” from a set of alternative ordered categories.^5^ Here, a series of single profiles are created based on a set of attributes and levels, with donors asked how frequently (e.g., twice a year, once a year, probably would not donate) they would donate according to the opportunities to donate. Frequency of donation estimated using this ordered categorical approach accurately predicted the actual donation frequency of the same donors.^7^ These predicted donation frequencies were combined with cost to evaluate the cost-effectiveness of options for changes to the blood service.^6^

These alternative forms of SP questions warrant careful consideration, as in health economic studies, such as those exploring the frequency of a behavior, this framing may be more suitable. However, there is little empirical work supporting the use of this form of SP question, in comparison with the considerable literature on DCEs.^8–10^ It is unknown whether the ordered categorical approach would provide similar estimates of relative preference to a DCE—the ordered categorical approach asks responders to state a preferred frequency according to a single profile, whereas a DCE asks the responder to make a distinct choice between profiles. While there is a growing literature that suggests different SP methods can produce different results,^11,12^ to our knowledge, no study has compared a DCE with an ordered categorical approach.

The aim of this article is to compare estimates of relative preferences from SP questions requiring ordered categorical versus discrete choice responses. To enable the comparison of preferences elicited between the 2 approaches, a common set of attributes and levels were formulated as choice tasks for the same sample (*N*= 8,933). The article proceeds as follows: the next section introduces the empirical example and provides a conceptual overview to distinguish the 2 ways of formulating the survey questions, the experimental design, sampling and analytical methods; the following section presents the results, and the fourth section discusses the findings and outlines areas for further research.

## Methods

### The Study

Eligible participants from the INTERVAL study (14,725 males and 14,006 females), a multicenter randomized controlled trial of alternative interdonation intervals,^13^ were invited via an email from NHS Blood and Transfusion (NHSBT) to participate in a web survey. After consenting to participate, donors were asked to provide information about the travel time for their last visit to donate whole blood before being asked to complete the 2 sets of SP questions. Donors’ baseline characteristics (gender, age, ethnicity, and blood type) and donation history (new donor or not, recruitment source, and the number of donations for the year prior to randomization) were extracted from the NHSBT donor database. Ethical approval was granted by NHS (reference 16/YH/0023) and LSHTM (reference 10384) Research Ethics Committees.

Attributes and levels were based on the previous study that used a literature review, input from policy makers at NHSBT, and qualitative research with blood donors.^6^ Five attributes were identified as pertaining to policy-relevant strategies for NHSBT: donor’s travel time, provision of a general health report (which might improve the experience of a donation visit), blood collection venue opening time, appointment availability, and the maximum number of annual donations (see Table 1). The maximum permitted donation frequency attribute is included to understand how donors might respond to increases in the maximum annual number of donations permitted. The annual limit may mean that some donors currently donate less frequently than they would like. The levels on this attribute differed for males (4 to 6) and females (3 to 4) because of gender differences in the minimum allowable time interval between donations (Table 1).

**Table 1:** Attributes and Levels for Males and Females in the Stated Preference Survey

Attribute	Levels	
Travel time	Your typical travel time	
	10 min shorter than your typical travel time	
	15 min longer than your typical travel time	
	30 min longer than your typical travel time	
Availability	Every day: Monday–Sunday	
	Every weekday: Monday–Friday	
	1 d every 2 mo: Monday–Friday	
	1 d every 2 mo: Saturday or Sunday	
Opening times	9 am–12 pm and 2 pm–5 pm	
	9 am–5 pm	
	9 am–8 pm	
	2 pm–8 pm	
Health report provided	No	
	Yes, after each donation	
Maximum permitted number of donations per year	Female	Male
	3 donations per year	4 donations per year
	4 donations per year	5 donations per year
		6 donations per year

**Figure 1:**
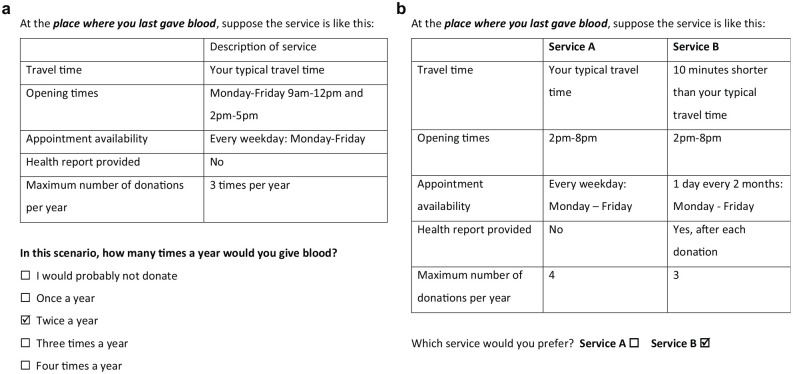
(a) Example of a stated preference–ordered categorical survey question. (b) Example of a discrete choice experiment survey question.

Female participants first answered ordered categorical questions with a single profile made up of 5 attributes and 5 response options (not donate to 4 times per year), and male participants ordered categorical questions, with 5 attributes and 7 response options (not donate to 6 times per year; see Figure 1a for an example ordered categorical question). Female and male participants then answered DCE questions with alternative profiles made up of 5 attributes, simply choosing which of the 2 profiles they preferred (see Figure 1b for an example DCE question).

An opt-out option was initially considered in the DCE but removed because the first Health Economics Modelling of Blood Donation Study (HEMO) survey reported that this option was chosen by less than 2% of respondents^6^ and the policy maker, NHSBT, did not authorize its inclusion. The order of the 2 SP tasks was not randomized to reduce the risk that donors might not complete all of the categorical responses questions required for the main policy evaluation. The survey questions were pretested and piloted prior to the main survey data collection.

### Experimental Design

Both the DCE and ordered categorical response tasks were generated with a D-efficient design created using Ngene™ 1.1.2 (Choice Metrics Pty Ltd, Sydney, Australia).^14^ The design included both main and interaction effects. D-efficiency is the most commonly used approach for generating efficient experimental design for choice experiments.^15^ The D-efficiency criterion satisfies the 4 principles of efficient design of a choice experiment: level balance, orthogonality, minimal overlap, and utility balance. A design that satisfies all of these principles leads to maximum D-efficiency.^16^ DCE response choice tasks used a generic A versus B design, and the ordered categorical aspect of the design was generated by including a utility function devoid of any attributes. The extra level in one of the attributes (maximum number of donations) for males rather than females meant that the number of choice sets differed by gender (36 for females and 72 for males). The choice sets were randomly allocated to respondents using a blocked design in Ngene, with each respondent asked to answer 7 choice tasks.

### Analysis

#### Modeling framework

Blood donors expressing a preference for one opportunity to donate over another (the DCE task) or deciding on how often to donate (SP-ordered categorial task) are assumed to make utility-maximizing choices given their preferences and the constraints they face. Theoretical foundations supporting the modeling approaches for DCE and SP-ordered categorical responses are included in the supplement (Supplementary 1). One way of summarizing donors’ preferences with respect to different attributes of the opportunities to donate is to estimate the marginal rate of substitution (MRS) between any pair of attributes.^17,18^ For DCEs, this information is derived from the coefficients of the different attributes estimated by modeling the choice of one option over another using the differences in the levels of the attributes. In the SP-ordered categorical case, the coefficients used to estimate the MRS are obtained by modeling the preferred frequency of donation as a function of the attribute levels that characterize a particular opportunity to donate. As the 2 approaches, of setting a task to indicate preferences or requiring the respondent to state donation frequency, are drawing from the same utility function, it could be expected that they would provide similar estimates of relative preference, according to, for example, the willingness to travel longer to attend a donor session in which a health report is provided. Alternatively, as the questions are framed in 2 different ways, it is conceivable that even if drawing from the same utility function, they could provide substantively different estimates of relative preference.

#### Modeling approach

We considered several regression models recommended for the analysis of SP data in health, recognizing the issues raised by the form of the respective response variables and assumptions made about the error terms. For each form of response, we reported measures of model fit across the alternative choices^19–21^ and the MRSs for travel time with respect to each of the other 4 attributes. The MRSs were calculated as the corresponding estimated regression coefficient divided by the negative (expected) estimated coefficient of travel time. Confidence intervals (CIs) for the MRSs were calculated by nonparametric bootstrapping with 100 replications. The mean differences in the MRS between the SP-ordered categorical and DCE approaches were tested using the normal approximation of bootstrap replicates, and *P* values were reported. All attribute levels were dummy coded except travel time. Travel time was considered a continuous variable, in which typical travel time was represented by the mean travel time (13 min) reported by survey respondents. For consistency with the survey design, the analysis was stratified by gender, recognizing that the maximum number of donations per year differed for male and female donors.

The first approach (model 1) specified a multinomial logit (MNL) for the DCE response and the generalized ordered-logit for the ordered categorical response.^20,22^ The MNL model assumed error terms are independent and identically distributed, which implies homogenous preferences across individuals. The generalized ordered-logit recognized the natural ordering in the donation frequency response variable (3 times per year, 2 times per year, etc.), to reflect the strength of preferences for a particular service configuration. The generalized ordered-logit model^23^ allowed for the impact of exogenous variables to affect the threshold parameters, thus relaxing the restrictive assumption of the traditional ordered discrete model, in which the effects of explanatory variables are restricted to be the same across dichotomized levels of the outcome variable.

The second approach (model 2) applied the MNL model to the SP-categorical response data and assumed that response variable was nominal (no ordering; model 2). This model allowed parameters to differ across the alternative attributes without imposing any restriction and offered greater flexibility and explanatory power, albeit at the expense of requiring more parameters to be estimated. Models 1 and 2 reported robust standard errors to recognize the correlation of individual-level responses.

We then extended the MNL for both responses to include random intercepts that allowed the parameter estimation to recognize the correlation in each individual’s responses across choice sets (model 3). To consider the potential impact of scale heterogeneity, we also considered a generalized multinomial logit (G-MNL), which allowed for the random error component to differ across individuals.^24^ We applied the G-MNL to the DCE data. The G-MNL could not be applied to the ordered categorical repsonse data, and so we applied an ordinal generalized linear model (GLM) that also allowed for scale heterogeneity.^22,25^

We considered the implications of imposing a forced choice for the DCE responses versus including an opt-out option for the ordered categorical responses, by reapplying model 3, as this was judged the most appropriate across the 2 forms of responses but after excluding respondents who chose the “I would probably not donate” option for all choice scenarios requiring the ordered response. While the main analyses assumed that the survey respondents were a random sample of the target population, we undertook sensitivity analyses, whereby the responses to both forms of survey question were weighted according to the characteristics of the target population of interest, defined by those of blood donors from the NHSBT register who were eligible because they had donated within the previous 12 mo. All analyses were conducted using Stata SE version 16.0 (StataCorp LLC, College Station, TX, USA).

## Results

The SP survey was completed by 4,179 females (29.8% response rate) and 4,754 males (32.3% response rate). Table 2 reports the characteristics of the analysis sample, those invited to complete the survey, and of eligible donors from the NHSBT register. Overall, in the analysis sample, 41% of men and 39% of women were in the age group 41–60 y, 14% of men and women were categorized as having high demand blood types, >90% of participants were self-defined as of White ethnic origin, and 90% of men and 85% of women had donated more than once in the past 12 mo. The ethnicity and blood type of the study sample were similar to all donors invited to complete the survey and those of the target population. A higher proportion of those who completed the surveys were older than 60 y and donated more frequently in the previous 12 mo compared with all of those surveyed and the NHSBT eligible donors (Table 2).

**Table 2:** Background Characteristics of Survey Respondents, Those Invited, and the NHSBT Eligible Population, *n* (%)

	Donors Who Responded to the Survey (Analysis Sample)		Donors Eligible for the Survey (All Invited)		NHSBT Eligible Population Who Donated in the Past 12 mo (Target Population)	
	Male (*n* = 4,754)	Female (*n* = 4,179)	Male (*n* = 14,725)	Female (*n* = 14,006)	Male (*n* = 353,736)	Female (*n* = 427,265)
Age group, y						
17–30	253 (5.32)	431 (10.31)	1,847 (12.54)	2,562 (18.29)	73,411 (20.75)	115,333 (26.99)
31–45	897 (18.87)	1,043 (24.96)	3,878 (26.34)	4,293 (30.65)	86,583 (24.47)	118,922 (27.83)
46–60	1,971 (41.46)	1,609 (38.50)	5,743 (39.00)	4,828 (34.47)	131,920 (37.29)	135,936 (31.82)
60+	1,633 (34.55)	1,096 (26.23)	3,257 (22.12)	2,323 (16.59)	61,849 (17.48)	57,074 (13.36)
Blood type^a^						
High demand	643 (13.53)	580 (13.88)	1,946 (13.22)	2,028 (14.48)	46,998 (13.29)	64,950 (15.20)
Standard demand	4,111 (86.47)	3,599 (86.12)	12,779 (86.78)	11,978 (85.52)	306,765 (86.71)	362,315 (85.80)
Ethnicity						
White	4,464 (93.90)	3,925 (93.92)	3,512 (91.76)	3,050 (93.17)	323,912 (91.56)	400,968 (93.85)
Black/mixed black	39 (0.82)	52 (1.24)	187 (1.27)	210 (1.50)	3,518 (0.99)	4,797 (1.12)
Asian/mixed Asian	106 (2.23)	76 (1.82)	494 (3.35)	286 (2.04)	12,677 (3.58)	9,050 (2.12)
Other or not stated	145 (3.05)	126 (3.02)	532 (3.61)	460 (3.28)	13,656 (3.86)	12,450 (2.91)
Number of donations in past 12 mo						
≤1	432 (9.09)	630 (15.08)	3,743 (25.42)	5,086 (36.31)	129,404 (36.58)	187,862 (43.97)
2–3	1,625 (34.18)	2,923 (69.94)	5,246 (35.63)	7,718 (55.10)	198,944 (56.24)	230,251 (53.89)
4+	2,697 (56.73)	626 (14.98)	5,736 (38.95)	1,202 (8.58)	25,415 (7.18)	9,152 (2.14)

a “Standard” demand blood types are O+, A+, B+, AB+, and AB− and “high” demand blood types are O−, A−, and B−.

Within the SP-ordered categorical response questions, a small proportion of participants (1.3% male and 1.1% female) always chose the opt-out, “I would probably not donate,” irrespective of the attribute levels. Within the DCE choice tasks requiring a binary response, there were no respondents whose choices were consistent with having lexicographic preferences. Lexicographic preferences were assessed by checking whether the level of a particular attribute always appeared to determine their choice. All of the SP questions were completed by 98.8% of male and 98.9% of female respondents for the questions requiring an ordered categorical response and 93.6% of male and 98.7% of female respondents for the DCE tasks.

For the SP-ordered categorical responses, the MNL (model 2) fitted the data better than the ordered logit (model 1) for both genders (Supplementary Table S2 and S4). For the ordered categorical responses, the inclusion of random effects was helpful in recognizing unexplained variation in responses across individuals, and therefore model 3 fitted best for both genders (Supplementary Tables S2 and S4). For the DCE response, there was unexplained variation at the level of the responder for females (ρ = 0.036) but not for males (ρ = 3.22 × 10^−6^), and so the inclusion of random effects improved model fit for women but not for men. The consideration of scale heterogeneity led to an improvement in model fit only in the response to the DCE questions for males (model 4 v. 3, Supplementary Table S1). For the ordered categorical responses, the ordinal GLM that considered scale heterogeneity led to worse fit for both genders (model 4 v. other models, Supplementary Tables S1 and S2).

The estimated coefficients from all the regression models were in line with prior expectations (see Appendix Tables S5–S8 for those from the best-fitting models). The estimated coefficients represent the effect of attribute levels on annual donation frequency for the ordered categorical questions and the probability of selecting a donation service with these particular attribute levels for the DCE response questions. The estimated coefficients suggest that for both forms of survey question, donors were willing to trade off increased travel time for a service change anticipated to improve their utility, the introduction of a health report, and for changes that would reduce constraints such as extending donor center opening hours or appointment availability or increasing the maximum number of donations permitted annually.

Table 3a,b presents the main results, the estimated MRSs from each model for travel time versus changes in the level of each of the categorical attributes for both sets of survey questions. The bootstrap estimates of MRSs are stable (see Supplementary Figures S1–S4 for the bootstrap trace of the best-fitting model). For example, respondents were only willing to trade off a small increase in travel time to receive a health report, irrespective of whether the choice was binary (DCE response; approximately 3 min of travel time) or from an ordered category (about 8 min). By contrast, if donors were to be offered less frequent appointments (1 d every 2 mo versus every day), they would require 30 min less travel time to compensate, whether the questions were framed to receive a binary or ordered categorical responses. The mean estimates of these MRSs were similar for both forms of survey questions across all the attributes for both genders and across all the models considered. The results were almost identical after excluding those respondents who choose to opt out for all the ordered categorical response scenarios (Supplementary Tables S9 and 10).

**Table 3a:** MRS Estimates (95% CI)^a^ for Each Categorical Survey Attribute versus Travel Time (in Minutes) for SP-Ordered Categorical and DCE Survey Responses from 4,757 Males, after Applying Alternative Regression Models^b^

	Model 1: Ordered Logit/MNL		Model 2: MNL		Model 3: MNL with Random Effects		Model 4: Ordinal GLM/G-MNL	
	SP-Ordered Categorical	DCE^c^	SP-Ordered Categorical	DCE^c^	SP-Ordered Categorical	DCE	SP-Ordered Categorical	DCE
Health report	2.79 [1.59, 4.00]	8.49 [7.13, 9.85]	2.74 [1.45, 4.04]	8.49 [7.13, 9.85]	2.67 [1.23, 4.10]	8.49 [7.47, 9.50]	2.42 [1.05, 3.79]	7.97 [7.00, 8.93]
Opening time: 9 am–5 pm*	0.78 [−0.95, 2.51]	1.98 [0.05, 3.91]	1.12 [−0.66, 2.91]	1.98 [0.05, 3.91]	2.34 [0.46, 4.21]	1.98 [0.46, 3.50]	0.77 [0.32, 1.22]	0.41 [−0.82, 1.94]
Opening time: 9 am–8 pm*	11.04 [9.15, 12.92]	13.83 [11.59, 16.07]	12.08 [10.15, 14.02]	13.83 [11.59, 16.07]	13.57 [11.33, 15.80]	13.83 [12.05, 15.61]	11.07 [9.42, 12.73]	7.65 [6.40, 9.46]
Opening time: 2 pm–8 pm*	5.50 [3.73, 7.28]	3.44 [1.31, 5.57]	5.79 [4.08, 7.51]	3.44 [1.31, 5.57]	6.64 [4.80, 8.48]	3.44 [2.00, 4.88]	5.58 [4.04, 7.13]	−0.68 [−2.17, 1.15]
Availability: Every weekday: Monday–Friday**	−6.57 [−8.75, −4.40]	−2.46 [−4.04, −0.88]	−7.48 [−9.60, −5.36]	−2.46 [−4.04, −0.88]	−7.99 [−10.45, −5.54]	−2.46 [−3.69, −1.24]	−4.63 [−6.04, −3.21]	−0.38 [−1.42, 0.58]
Availability: 1 d every 2 mo: Monday–Friday**	−16.06 [−18.15, −13.97]	−18.31 [−20.57, −16.05]	−17.13 [−19.24, −15.02]	−18.31 [−20.57, −16.05]	−16.52 [−18.75, −14.29]	−18.31 [−19.79, −16.84]	−14.25 [−16.32, −12.18]	−15.63 [−17.24, −14.55]
Availability: 1 d every 2 mo: Saturday or Sunday**	−28.01 [−30.39, −25.63]	−33.65 [−36.55, −30.75]	−28.92 [−31.22, −26.62]	−33.65 [−36.55, −30.75]	−30.73 [−33.26, −28.19]	−33.65 [−35.46, −31.84]	−29.14 [−30.74, −27.55]	−32.16 [−34.17, −30.25]
Max donations: 5***	13.69 [11.89, 15.49]	11.81 [9.99, 13.63]	12.78 [10.92, 14.64]	11.81 [9.99, 13.63]	7.44 [5.48, 9.39]	11.81 [10.39, 13.23]	18.17 [16.36, 19.99]	10.48 [9.16, 12.03]
Max donations: 6***	23.20 [21.31, 25.10]	17.72 [15.88, 19.56]	21.83 [19.90, 23.75]	17.72 [15.88, 19.56]	7.54 [5.64, 9.43]	17.72 [16.44, 19.00]	36.74 [33.87, 39.61]	16.20 [14.74, 17.70]

CI, confidence interval; DCE, discrete choice experiment; GLM, generalized linear model; G-MNL, generalized multinomial logit; MNL, multinomial logit; MRS, marginal rates of substitution; NHSBT, NHS Blood and Transfusion; SP, stated preference.

a Confidence interval calculated with nonparametric bootstrap.

b Reference category: *9 am–12 pm and 2 pm–5 pm; **every day: Monday to Sunday; ***4 donations per year.

c Models 1 and 2 are the same for the DCE model.

**Table 3b:** MRS Estimates (95% CI)^a^ for Each Categorical Survey Attribute versus Travel Time (in min) for SP-Ordered Categorical and DCE Survey Responses from 4,179 Females, after Applying Alternative Regression Models^b^

	Model 1: Ordered logit/MNL^c^		Model 2: MNL^c^		Model 3: MNL with Random Effects		Model 4: Ordinal GLM/G-MNL	
	SP-Ordered Categorical	DCE	SP-Ordered Categorical	DCE	SP-Ordered Categorical	DCE	SP-Ordered Categorical	DCE
Health report	3.73 [2.55, 4.90]	11.42 [9.84, 13.00]	3.89 [2.68, 5.10]	11.42 [9.84, 13.00]	3.24 [1.96, 4.52]	11.29 [10.31, 12.27]	3.57 [2.75, 4.39]	11.44 [10.30, 12.51]
Opening time: 9 am–5 pm*	3.53 [1.89, 5.16]	13.09 [11.41, 14.77]	3.19 [1.59, 4.79]	13.09 [11.41, 14.77]	4.01 [2.40, 5.61]	12.84 [11.79, 13.89]	4.28 [2.96, 5.61]	13.03 [11.97, 14.40]
Opening time: 9 am–8 pm*	15.46 [13.54, 17.38]	22.19 [20.24, 24.14]	15.46 [13.56, 17.36]	22.19 [20.24, 24.14]	18.17 [16.01, 20.34]	21.92 [20.73, 23.12]	16.59 [16.16, 17.01]	22.24 [21.00, 23.88]
Opening time: 2 pm–8 pm*	8.32 [6.49, 10.14]	8.59 [6.89, 10.29]	8.45 [6.72, 10.19]	8.59 [6.89, 10.29]	11.01 [9.00, 13.03]	8.46 [7.23, 9.68]	9.02 [8.25, 9.80]	8.45 [7.10, 10.04]
Availability: every weekday: Monday–Friday **	−9.58 [−11.96, −7.20]	−2.80 [−4.54, −1.05]	−10.36 [−12.28, −8.44]	−2.80 [−4.54, −1.05]	−9.75 [−12.07, −7.44]	−2.71 [−3.99, −1.43]	7.74 [−8.79, −6.70]	−2.76 [−4.01, −1.52]
Availability: 1 d every 2 mo: Monday–Friday**	−21.19 [−23.39, −18.99]	−21.61 [−23.80, −19.42]	−21.67 [−23.65, −19.69]	−21.61 [−23.80, −19.42]	−19.75 [−22.07, −17.43]	−21.56 [−23.06, −20.07]	−20.05 [−21.62, −18.48]	−21.57 [−23.08, −20.43]
Availability: 1 d every 2 mo: Saturday or Sunday**	−27.75 [−30.22, −25.29]	−29.05 [−31.08, −27.02]	−27.95 [−30.17, −25.73]	−29.05 [−31.08, −27.02]	−26.38 [−28.92, −23.85]	−28.92 [−30.17, −27.66]	−29.40 [−30.70, −28.09]	−29.19 [−30.72, −27.58]
Max donations: 4***	14.49 [13.19, 15.79]	11.75 [10.57, 12.93]	13.19 [11.96, 14.41]	11.75 [10.57, 12.93]	4.23 [2.73, 5.73]	11.68 [10.91, 12.46]	20.84 [19.64, 22.04]	12.08 [10.98, 12.97]

CI, confidence interval; DCE, discrete choice experiment; GLM, generalized linear model; G-MNL, generalized multinomial logit; MNL, multinomial logit; MRS, marginal rates of substitution; SP, stated preference.

a Confidence interval calculated with nonparametric bootstrap.

b Reference category: *9 am–12 pm and 2 pm–5 pm; **every day: Monday to Sunday; ***3 donations per year.

c Models 1 and 2 are the same for the DCE model.

**Figure 2:**
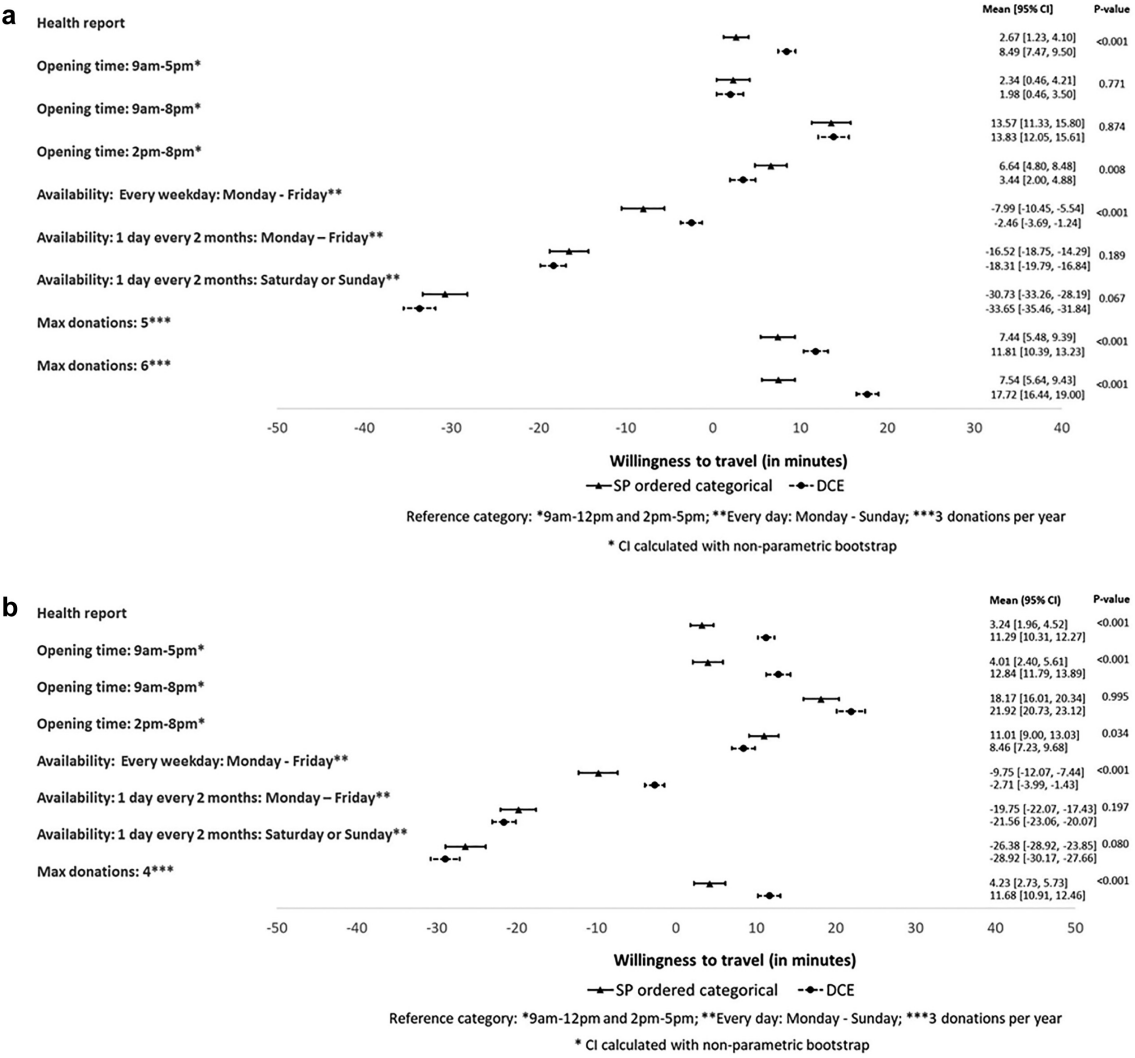
(a) Marginal rates of substitution from SP-ordered categorical and DCE survey responses for 4,754 males. (b) Marginal rates of substitution from SP-ordered categorical and DCE survey responses for 4,179 females. DCE, discrete choice experiment; SP, stated preference.

Figure 2a,b presents the MRS estimates from model 3 for travel time versus changes in each of the other blood service attributes, for both sets of survey questions. The differences between the SP-ordered categorical and DCE approaches in the estimates of the mean MRSs were statistically significant at the 5% level for some attributes and levels, for example, introduction of the health report (both genders), allowing appointment availability every weekday, changing opening time to 2 pm to 8 pm (males) or 9 am to 5 pm (females) or increasing the maximum number of donations permitted (both genders). For the other service attributes, the differences in the estimated mean MRSs were not statistically significant. However, across all attributes and levels, the mean differences in the estimated MRSs between the survey approaches were relatively small. The maximum difference in the estimated MRSs between the different forms of survey questions was 10 min (for the attribute that increases the maximum donation frequency to 6 for males), but the mean difference was 5 min or less for all other attributes for males and 8 min or less for females. The estimated MRSs after reweighting the estimates according to the observed characteristics of the target population were similar to those from the unweighted survey population (Appendix Tables S11–S12).

## Discussion

This article compares overall (full sample) preferences elicited from 2 different SP tasks. These approaches were contrasted within the same survey administered to a large sample of blood donors. It was recognized a priori that these 2 ways of formulating the survey questions could lead to different estimates of relative preference, as they ask different questions, albeit drawing from the same utility function. However, the 2 forms of questions provided similar estimates of MRSs between a “cost” (increased travel time) and factors anticipated to increase utility (e.g., introduction of a health report) or a relax constraints (e.g., extended donor centre opening hours). The mean differences between the ordered-categorical and discrete-choice response approaches in the estimated MRSs for travel time versus all other attributes did not exceed 10 min for all models that provided reasonable fit to the data. It is unlikely that these small differences would imply differential policy recommendations according to the form of choice task within the SP survey. The estimated MRSs were similar across regression approaches.

This study exemplifies how SP survey questions can be formulated to provide responses on an ordered categorical scale required for the parameter of interest (donation frequency), as well as according to more traditional DCE tasks. The finding that any differences in the estimated MRSs are not of substantive importance offers some reassurance for policy makers in that estimates of relative preference may be robust to alternative ways of framing the survey questions. It should also be recognized that this robustness may reflect some of the study’s strengths, in that the sample size was relatively large compared with previous SP surveys in health, differences attributable to unobserved heterogeneity were minimized by the same respondents completing both sets of survey questions, and the same analytical model was found to fit both forms of response data relatively well.

Considerable literature has found inconsistent results in comparing different types of SP tasks.^26–31^ Such comparisons are fraught with challenges. In particular, previous attempts have faced major methodological concerns about unobserved differences (heterogeneity) between the comparison groups, inadequate sample sizes, and the absence of a metric for comparison across the alternative designs.^32^ Moreover, most studies have not been able to assess the predictive accuracy of the SP estimates with revealed preference data, and so while differences between approaches might be identified, it is impossible to determine which approach is more accurate. The study by Ryan and Watson is one notable exception; here, they compared stated screening uptake with both a contingent valuation and DCE and compared with actual uptake.^29^ They found signiﬁcant differences in the stated screening intention between both methods and with actual screening uptake. This contrasts to this study, which finds similar results between approaches and with observed blood donation frequency.

This article has some limitations and provokes several areas of future research. First, although the MRS estimates from the SP-ordered categorical questions and DCE tasks were similar, they were not directly compared with revealed preference data.^33^ However, the donation frequency predicted from the SP-ordered categorical survey questions was previously found to be similar to observed donation frequency.^7^ Second, the article exemplifies the use of an SP-ordered categorical versus a DCE task, within a single application. It is unknown whether in other settings there are substantive differences across approaches and how these should be presented in policy recommendations. Third, the questions requiring an ordered categorical response included an opt-out option, whereas the DCE tasks did not. However, this is unlikely to have materially affected the findings, since only a small proportion (<2%) of donors indicated a preference to not donate across all the ordered categorical choice sets received, and the exclusion of these individuals from the analysis had a negligible impact on the results. Fourth, the SP-ordered categorical versus DCE tasks were not presented in random order. The SP-ordered categorical questions were always presented first, and this may have led to the lower completion rate for the DCE tasks for males. Nonrandomized ordering of choice tasks may lead to strategic behavior^34^ and therefore may bias the MRS estimates. However, there was no evidence of strategic or nontrading behavior across the survey formats for either gender. Finally, the analysis models did not fully consider preference heterogeneity, as that was beyond the immediate scope of the article. While the G-MNL has the flexibility to simultaneously address individual-specific scale and preference heterogeneity for DCE response data, the application to ordered categorical responses requires extension.^24^ For the DCE responses, we have assessed the preference heterogenity using latent class analysis but did not find any evidence that preferences from DCE responses are obscured by preference heterogenity (Supplement 2).

Future studies that contrast alternative ways of framing SP questions would be seful, and should consider whether there is additional cognitive burden associated with requiring ordered categorical versus DCE choice tasks. If so, it would be useful to consider whether the parameter of prime policy interest could still be provided but with questions in a simpler form.^35^ Future research could also extend existing choice models^24,25^ to evaluate the impact of alternative forms of survey question for both overall (full sample) preferences and preferences across subgroups in the presence of preference and scale heterogeneity.^36–38^

In conclusion, this article highlights the potential value of alternative choice framings, rather than relying solely on DCEs, and encourages researchers to consider more carefully the most suitable framing of choices in SP studies. The decision of which choice task is most appropriate for a given study depends on many factors and can incur a tradeoff between theoretical underpinning and the best way to frame a task that responders find both realistic and understandable.

## Supplemental Material

sj-docx-1-mdm-10.1177_0272989X221145048 – Supplemental material for A Comparison of Ordered Categorical versus Discrete Choices within a Stated Preference Survey of Whole-Blood Donors Click here for additional data file. Supplemental material, sj-docx-1-mdm-10.1177_0272989X221145048 for A Comparison of Ordered Categorical versus Discrete Choices within a Stated Preference Survey of Whole-Blood Donors by Zia Sadique, John Cairns, Kaat De Corte, Sarah Willis, Alec Miners, Nick Bansback and Richard Grieve in Medical Decision Making

sj-docx-2-mdm-10.1177_0272989X221145048 – Supplemental material for A Comparison of Ordered Categorical versus Discrete Choices within a Stated Preference Survey of Whole-Blood Donors Click here for additional data file. Supplemental material, sj-docx-2-mdm-10.1177_0272989X221145048 for A Comparison of Ordered Categorical versus Discrete Choices within a Stated Preference Survey of Whole-Blood Donors by Zia Sadique, John Cairns, Kaat De Corte, Sarah Willis, Alec Miners, Nick Bansback and Richard Grieve in Medical Decision Making

sj-docx-3-mdm-10.1177_0272989X221145048 – Supplemental material for A Comparison of Ordered Categorical versus Discrete Choices within a Stated Preference Survey of Whole-Blood Donors Click here for additional data file. Supplemental material, sj-docx-3-mdm-10.1177_0272989X221145048 for A Comparison of Ordered Categorical versus Discrete Choices within a Stated Preference Survey of Whole-Blood Donors by Zia Sadique, John Cairns, Kaat De Corte, Sarah Willis, Alec Miners, Nick Bansback and Richard Grieve in Medical Decision Making

## References

[1] Ryan M Gerard K Amaya-Amaya M . Using Discrete Choice to Value Health and Health Care. Dordrecht (the Netherlands): Springer; 2008.

[2] Adamowicz W Boxall P Williams M Louviere J . Stated preference approaches for measuring passive use values: choice experiments and contingent valuation. American Journal of Agricultural Economics. 1998;80(1):64–75.

[3] Hanemann WM . Welfare evaluations in contingent valuation experiments with discrete responses. American Journal of Agricultural Economics. 1984;66(3):332–41.

[4] Soekhai V de Bekker-Grob EW Ellis AR Vass CM . Discrete choice experiments in health economics: past, present and future. Pharmacoeconomics. 2019;37(2):201–26. 10.1007/s40273-018-0734-2 PMC6386055 30392040

[5] Carson RT Louviere JJ . A common nomenclature for stated preference elicitation approaches. Environmental and Resource Economics. 2011;49(4):539–59.

[6] Grieve R Willis S De Corte K , et al. Options for possible changes to the blood donation service: health economics modelling. Health Services and Delivery Research. No. 6.40. Southampton (UK): NIHR Journals Library; 2018. 30540399

[7] de Corte K Cairns J Grieve R . Stated versus revealed preferences: an approach to reduce bias. Health Econ. 2021;30(5):1095–123. 10.1002/hec.4246 33690931

[8] Ali S Ronaldson S . Ordinal preference elicitation methods in health economics and health services research: using discrete choice experiments and ranking methods. Br Med Bull. 2012;103(1):21–44. 22859714 10.1093/bmb/lds020

[9] Kind P. Applying paired comparisons models to EQ-5D valuations-deriving TTO utilities from ordinal preference data. In: Kind P Brooks R Rabin R , eds. EQ-5D Concepts and Methods: A Developmental History. New York: Springer; 2005. p 201–20.

[10] Ryan M Hughes J . Using conjoint analysis to assess women’s preferences for miscarriage management. Health Econ. 1997;6(3):261–73. 10.1002/(sici)1099-1050(199705)6:3<261::aid-hec262>3.0.co;2-n 9226144

[11] Jacobsen JB Thorsen BJ Dubgaard A , eds. Sensitivity to scale in stated preference valuation methods: a comparison of methods based on valuation of heath in Denmark. In: Scandinavian Forest Economics: Proceedings of the Biennial Meeting of the Scandinavian Society of Forest Economics. Uppsala, Sweden: Scandinavian Society of Forest Economics; 2006.

[12] Lancsar E Louviere J Flynn T . Several methods to investigate relative attribute impact in stated preference experiments. Soc Sci Med. 2007;64(8):1738–53. 10.1016/j.socscimed.2006.12.007 17257725

[13] Di Angelantonio E Thompson SG Kaptoge S , et al. Efficiency and safety of varying the frequency of whole blood donation (INTERVAL): a randomised trial of 45 000 donors. Lancet. 2017;390(10110):2360–71. 10.1016/S0140-6736(17)31928-1 PMC5714430 28941948

[14] Bridges JF Hauber AB Marshall D , et al. Conjoint analysis applications in health—a checklist: a report of the ISPOR Good Research Practices for Conjoint Analysis Task Force. Value Health. 2011;14(4):403–13. 10.1016/j.jval.2010.11.013 21669364

[15] Street DJ Burgess L . The Construction of Optimal Stated Choice Experiments: Theory and Methods. New York: John Wiley & Sons; 2007.

[16] Carlsson F Martinsson P . Design techniques for stated preference methods in health economics. Health Econ. 2003;12(4):281–94. 10.1002/hec.729 12652515

[17] Lancsar E Louviere J Flynn T . Several methods to investigate relative attribute impact in stated preference experiments. Soc Sci Med. 2007;64(8):1738–53. 10.1016/j.socscimed.2006.12.007 17257725

[18] Ryan M . A comparison of stated preference methods for estimating monetary values. Health Econ. 2004;13(3):291–6. 10.1002/hec.818 14981653

[19] de Bekker-Grob EW Ryan M Gerard K . Discrete choice experiments in health economics: a review of the literature. Health Econ. 2012;21(2):145–72. 10.1002/hec.1697 22223558

[20] Hauber AB Gonzalez JM Groothuis-Oudshoorn CG , et al. Statistical methods for the analysis of discrete choice experiments: a report of the ISPOR Conjoint Analysis Good Research Practices Task Force. Value Health. 2016;19(4):300–15. 10.1016/j.jval.2016.04.004 27325321

[21] Lancsar E Fiebig DG Hole AR . Discrete choice experiments: a guide to model specification, estimation and software. Pharmacoeconomics. 2017;35(7):697–716. 28374325 10.1007/s40273-017-0506-4

[22] Williams R . OGLM: Stata Module to Estimate Ordinal Generalized Linear Models. Statistical Software Components S453402, Boston College Department of Economics; 2016.

[23] Williams R . Generalized ordered logit/partial proportional odds models for ordinal dependent variables. Stata J. 2006;6(1):58–82.

[24] Fiebig DG Keane MP Louviere J Wasi N . The generalized multinomial logit model: accounting for scale and coefficient heterogeneity. Market Sci. 2010;29(3):393–421.

[25] Williams R . Fitting heterogeneous choice models with oglm. Stata J. 2010;10(4):540–67.

[26] Carson RT Flores NE Meade NF . Contingent valuation: controversies and evidence. Environ Res Econ. 2001;19(2):173–210.

[27] Desvousges WH Smith VK Fisher A . Option price estimates for water quality improvements: a contingent valuation study for the Monongahela River. J Environ Econ Manage. 1987;14(3):248–67.

[28] Hanley N MacMillan D Wright RE , et al. Contingent valuation versus choice experiments: estimating the benefits of environmentally sensitive areas in Scotland. Journal of Agricultural Economics. 1998;49(1):1–15.

[29] Ryan M Watson V . Comparing welfare estimates from payment card contingent valuation and discrete choice experiments. Health Econ. 2009;18(4):389–401. 18677721 10.1002/hec.1364

[30] Yoo HI Doiron D . The use of alternative preference elicitation methods in complex discrete choice experiments. J Health Econ. 2013;32(6):1166–79. 10.1016/j.jhealeco.2013.09.009 24144729

[31] Huls SP Lancsar E Donkers B Ride J . Two for the price of one: if moving beyond traditional single-best discrete choice experiments, should we use best-worst, best-best or ranking for preference elicitation? Health Econ. 2022;31(12):2630–47. 10.1002/hec.4599 PMC9826006 36102864

[32] Benjamin DJ Heffetz O Kimball MS Rees-Jones A . Can marginal rates of substitution be inferred from happiness data? evidence from residency choices. Am Econ Rev. 2014;104(11):3498–528. 10.1257/aer.104.11.3498 PMC4231441 25404759

[33] de Bekker-Grob EW Donkers B Bliemer MCJ Veldwijk J Swait JD . Can healthcare choice be predicted using stated preference data? Soc Sci Med. 2020;246:112736. 10.1016/j.socscimed.2019.112736 31887626

[34] Nguyen TC Le HT Nguyen HD Ngo MT Nguyen HQ . Examining ordering effects and strategic behaviour in a discrete choice experiment. Econ Anal Policy. 2021;70:394–413.

[35] Flynn TN Bilger M Malhotra C Finkelstein EA . Are efficient designs used in discrete choice experiments too difficult for some respondents? A case study eliciting preferences for end-of-life care. Pharmacoeconomics. 2016;34(3):273–84. 10.1007/s40273-015-0338-z 26589411

[36] Hess S Train K . Correlation and scale in mixed logit models. J Choice Model. 2017;23:1–8.

[37] Vass CM Wright S Burton M Payne K . Scale heterogeneity in healthcare discrete choice experiments: a primer. Patient. 2018;11(2):167–73. 10.1007/s40271-017-0282-4 29032437

[38] Vass C Boeri M Karim S , et al. Accounting for preference heterogeneity in discrete-choice experiments: an ISPOR special interest group report. Value Health. 2022;25(5):685–94. 10.1016/j.jval.2022.01.012 35500943

