# Supplementary material for: A Comparison of Ordered Categorical versus Discrete Choices within a Stated Preference Survey of Whole-Blood Donors
Source: Med Decis Making. 2022 Dec 24;43(3):362–73. doi: 10.1177/0272989X221145048 (PMC10021117; doi:10.1177/0272989X221145048)
Supplement: sj-docx-1-mdm-10.1177_0272989X221145048 – Supplemental material for A Comparison of Ordered Categorical versus Discrete Choices within a Stated Preference Survey of Whole-Blood Donors [file sj-docx-1-mdm-10.1177_0272989X221145048.docx]

**Table S1. Model fits of alternative regression models applied to DCE survey responses (males)**

|  | **Model 1: MNL** | **Model 2: MNL** | **Model 3:**  **MNL with random effects** | **Model 4:**  **G-MNL** |
| --- | --- | --- | --- | --- |
| **Log-likelihood** | -18594 | -18594 | -18594 | -18353 |
| **AIC** | 37211 | 37211 | 37213 | 36730 |
| **BIC** | 37311 | 37311 | 37313 | 36839 |

**Table S2. Model fits of alternative regression models applied to SP-ordered categorical survey responses (males)**

|  | **Model 1:**  **Ordered logit** | **Model 2:**  **MNL** | **Model 3:**  **MNL with random effects** | **Model 4:**  **GLM allowing for scale heterogeneity** |
| --- | --- | --- | --- | --- |
| **Log-likelihood** | -41101 | -41049 | -38894 | -44053 |
| **AIC** | 82314 | 82229 | 77921 | 88159 |
| **BIC** | 82776 | 82229 | 78474 | 88373 |

**Table S3. Model fit of alternative regression models applied to DCE survey responses (females)**

|  | **Model 1:**  **MNL** | **Model 2:**  **MNL** | **Model 3:**  **MNL with random effects** | **Model 4:**  **G-MNL** |
| --- | --- | --- | --- | --- |
| **Log-likelihood** | -15511 | -15511 | -15494 | -15499 |
| **AIC** | 31043 | 31043 | 31010 | 31020 |
| **BIC** | 31133 | 31133 | 31100 | 31119 |

**Table S4. Model fit of alternative regression models applied to SP-ordered categorical survey responses (females)**

|  | **Model 1:**  **Ordered logit** | **Model 2:**  **MNL** | **Model 3:**  **MNL with random effects** | **Model 4:**  **GLM allowing for scale heterogeneity** |
| --- | --- | --- | --- | --- |
| **Log-likelihood** | -31070 | -31006 | -29313 | -31705 |
| **AIC** | 62214 | 62092 | 58708 | 63455 |
| **BIC** | 62515 | 62417 | 59041 | 63633 |

**Table S5. Results from random effects multinomial logit regression model (model 3) – SP-ordered categorical survey responses for males**

|  |  | **b** | **SE** |
| --- | --- | --- | --- |
| >=1 donation | Travel time (minutes) | -0.0389 | 0.0039 |
|  | Health report | 0.0644 | 0.1059 |
|  | Opening times: 9am-5pm | 0.1413 | 0.1349 |
|  | Opening times: 9am-8pm | 0.1903 | 0.1605 |
|  | Opening times: 2pm-8pm | -0.0029 | 0.1479 |
|  | Availability: Every weekday: Monday - Friday | -0.3297 | 0.1894 |
|  | Availability: 1 day every 2 months: Monday - Friday | -0.2215 | 0.1694 |
|  | Availability: 1 day every 2 months: Saturday or Sunday | -0.9003 | 0.1660 |
|  | Maximum donations: 5 donations per year | -0.0378 | 0.1279 |
|  | Maximum donations: 6 donations per year | -0.0469 | 0.1282 |
| >=2 donations | Travel time (minutes) | -0.0305 | 0.0026 |
|  | Health report | 0.1300 | 0.0683 |
|  | Opening times: 9am-5pm | 0.2561 | 0.0930 |
|  | Opening times: 9am-8pm | 0.7742 | 0.1000 |
|  | Opening times: 2pm-8pm | 0.4752 | 0.0947 |
|  | Availability: Every weekday: Monday - Friday | -0.2175 | 0.1121 |
|  | Availability: 1 day every 2 months: Monday - Friday | -0.3943 | 0.1063 |
|  | Availability: 1 day every 2 months: Saturday or Sunday | -1.1255 | 0.1049 |
|  | Maximum donations: 5 donations per year | -0.0151 | 0.0823 |
|  | Maximum donations: 6 donations per year | -0.0385 | 0.0825 |
| >=3 donations | Travel time (minutes) | -0.0516 | 0.0025 |
|  | Health report | 0.0865 | 0.0655 |
|  | Opening times: 9am-5pm | 0.0913 | 0.0895 |
|  | Opening times: 9am-8pm | 0.7698 | 0.0949 |
|  | Opening times: 2pm-8pm | 0.4347 | 0.0900 |
|  | Availability: Every weekday: Monday - Friday | -0.5337 | 0.1013 |
|  | Availability: 1 day every 2 months: Monday - Friday | -0.8971 | 0.0974 |
|  | Availability: 1 day every 2 months: Saturday or Sunday | -1.7732 | 0.0970 |
|  | Maximum donations: 5 donations per year | 0.2123 | 0.0792 |
|  | Maximum donations: 6 donations per year | 0.1313 | 0.0799 |
| >=4 donations | Travel time (minutes) | -0.0719 | 0.0021 |
|  | Health report | 0.1669 | 0.0526 |
|  | Opening times: 9am-5pm | 0.1814 | 0.0707 |
|  | Opening times: 9am-8pm | 0.9859 | 0.0766 |
|  | Opening times: 2pm-8pm | 0.4282 | 0.0726 |
|  | Availability: Every weekday: Monday - Friday | -0.5013 | 0.0816 |
|  | Availability: 1 day every 2 months: Monday - Friday | -1.2209 | 0.0800 |
|  | Availability: 1 day every 2 months: Saturday or Sunday | -2.2239 | 0.0798 |
|  | Maximum donations: 5 donations per year | -1.0221 | 0.0639 |
|  | Maximum donations: 6 donations per year | -0.9843 | 0.0633 |
| >=5 donations | Travel time (minutes) | -0.0793 | 0.0023 |
|  | Health report | 0.1990 | 0.0572 |
|  | Opening times: 9am-5pm | 0.0953 | 0.0780 |
|  | Opening times: 9am-8pm | 1.0065 | 0.0828 |
|  | Opening times: 2pm-8pm | 0.5088 | 0.0792 |
|  | Availability: Every weekday: Monday - Friday | -0.6301 | 0.0870 |
|  | Availability: 1 day every 2 months: Monday - Friday | -1.3496 | 0.0858 |
|  | Availability: 1 day every 2 months: Saturday or Sunday | -2.4439 | 0.0860 |
|  | Maximum donations: 5 donations per year | 2.6851 | 0.0811 |
|  | Maximum donations: 6 donations per year | 0.8385 | 0.0886 |
| 6 donations | Travel time (minutes) | -0.0823 | 0.0022 |
|  | Health report | 0.2625 | 0.0548 |
|  | Opening times: 9am-5pm | 0.2029 | 0.0744 |
|  | Opening times: 9am-8pm | 1.0685 | 0.0798 |
|  | Opening times: 2pm-8pm | 0.4999 | 0.0761 |
|  | Availability: Every weekday: Monday - Friday | -0.6549 | 0.0840 |
|  | Availability: 1 day every 2 months: Monday - Friday | -1.3885 | 0.0829 |
|  | Availability: 1 day every 2 months: Saturday or Sunday | -2.4049 | 0.0828 |
|  | Maximum donations: 5 donations per year | 0.1246 | 0.0738 |
|  | Maximum donations: 6 donations per year | 1.7123 | 0.0673 |

b=coefficient, SE=standard error

**Table S6. Results from random effects multinomial logit regression model (model 3) – SP-ordered categorical survey responses for females**

|  |  | **b** | **SE** |
| --- | --- | --- | --- |
| >=1 donation | Travel time (minutes) | -0.0417 | 0.0028 |
|  | Health report | 0.0721 | 0.0728 |
|  | Opening times: 9am-5pm | 0.0701 | 0.0967 |
|  | Opening times: 9am-8pm | 0.6503 | 0.1061 |
|  | Opening times: 2pm-8pm | 0.3268 | 0.1005 |
|  | Availability: Every weekday: Monday - Friday | -0.2804 | 0.1263 |
|  | Availability: 1 day every 2 months: Monday - Friday | -0.3879 | 0.1191 |
|  | Availability: 1 day every 2 months: Saturday or Sunday | -0.6621 | 0.1168 |
|  | Maximum donations: 4 donations per year | -0.0828 | 0.0730 |
| >=2 donations | Travel time (minutes) | -0.0390 | 0.0023 |
|  | Health report | 0.1748 | 0.0597 |
|  | Opening times: 9am-5pm | 0.3499 | 0.0826 |
|  | Opening times: 9am-8pm | 1.1248 | 0.0883 |
|  | Opening times: 2pm-8pm | 0.8065 | 0.0834 |
|  | Availability: Every weekday: Monday - Friday | -0.4321 | 0.0967 |
|  | Availability: 1 day every 2 months: Monday - Friday | -0.7191 | 0.0941 |
|  | Availability: 1 day every 2 months: Saturday or Sunday | -1.0413 | 0.0923 |
|  | Maximum donations: 4 donations per year | 0.1303 | 0.0599 |
| >=3 donations | Travel time (minutes) | -0.0746 | 0.0020 |
|  | Health report | 0.2085 | 0.0491 |
|  | Opening times: 9am-5pm | 0.2406 | 0.0671 |
|  | Opening times: 9am-8pm | 1.2908 | 0.0725 |
|  | Opening times: 2pm-8pm | 0.7459 | 0.0683 |
|  | Availability: Every weekday: Monday - Friday | -0.7553 | 0.0761 |
|  | Availability: 1 day every 2 months: Monday - Friday | -1.5536 | 0.0771 |
|  | Availability: 1 day every 2 months: Saturday or Sunday | -2.1112 | 0.0759 |
|  | Maximum donations: 4 donations per year | -0.9753 | 0.0504 |
| >=4 donations | Travel time (minutes) | -0.0869 | 0.0021 |
|  | Health report | 0.2985 | 0.0505 |
|  | Opening times: 9am-5pm | 0.3194 | 0.0694 |
|  | Opening times: 9am-8pm | 1.4470 | 0.0744 |
|  | Opening times: 2pm-8pm | 0.8588 | 0.0705 |
|  | Availability: Every weekday: Monday - Friday | -0.8327 | 0.0771 |
|  | Availability: 1 day every 2 months: Monday - Friday | -1.7902 | 0.0786 |
|  | Availability: 1 day every 2 months: Saturday or Sunday | -2.2894 | 0.0776 |
|  | Maximum donations: 5 donations per year | 1.4173 | 0.0525 |

b=coefficient, SE=standard error

**Table S7. Results from random effects multinomial logit regression model (model 3) – DCE survey responses for males**

|  | | **b** | **SE** |
| --- | --- | --- | --- |
| Travel time (minutes) | | - 0.038 | 0.001 |
| Health report | | 0.322 | 0.020 |
| Opening times | |  |  |
|  | 9am-5pm | 0.075 | 0.028 |
|  | 9am-8pm | 0.525 | 0.032 |
|  | 2pm-8pm | 0.131 | 0.031 |
| Availability | |  |  |
|  | Every weekday: Monday - Friday | -0.093 | 0.026 |
|  | 1 day every 2 months: Monday - Friday | -0.695 | 0.028 |
|  | 1 day every 2 months: Saturday or Sunday | -1.278 | 0.032 |
| Maximum donations | |  |  |
|  | 5 donations per year | 0.448 | 0.027 |
|  | 6 donations per year | 0.673 | 0.027 |
| Log likelihood | | -18594.489 |  |
| AIC | | 37210.980 |  |
| BIC | | 37310.660 |  |

b=coefficient, SE=standard error

**Table S8. Results from random effects multinomial logit regression model (model 3) – DCE survey responses for females**

|  | | **b** | **SE** |
| --- | --- | --- | --- |
| Travel time (minutes) | | -0.048 | 0.001 |
| Health report | | 0.544 | 0.027 |
| Opening times | |  |  |
|  | 9am-5pm | 0.624 | 0.030 |
|  | 9am-8pm | 1.058 | 0.032 |
|  | 2pm-8pm | 0.410 | 0.033 |
| Availability | |  |  |
|  | Every weekday: Monday - Friday | -0.133 | 0.033 |
|  | 1 day every 2 months: Monday - Friday | -1.030 | 0.035 |
|  | 1 day every 2 months: Saturday or Sunday | -1.385 | 0.030 |
| Maximum donations - 4 donations per year | | 0.560 | 0.023 |
| Log likelihood | | -15511.485 |  |
| AIC | | 31042.970 |  |
| BIC | | 31132.530 |  |

b=coefficient, SE=standard error

**Table S9. MRS estimates (95% CI)* for each categorical survey attribute versus travel time for SP-ordered categorical and DCE survey responses for males for the full sample and after excluding opt-out respondents.**

|  | **Model 3:**  **MNL with random effects**  **(full sample)** | | **Model 3:**  **MNL with random effects**  **(excluding opt-out respondents)** | |
| --- | --- | --- | --- | --- |
|  | **SP-ordered**  **Categorical** | **DCE** | **SP-ordered**  **categorical** | **DCE** |
| Health report | 2.67  [1.23, 4.10] | 8.49  [7.47, 9.50] | 2.70  [1.22, 4.18] | 8.49  [7.47, 9.50] |
| Opening time: 9am-5pm * | 2.34  [0.46, 4.21] | 1.98  [0.46, 3.50] | 2.40  [0.05, 4.75] | 1.98  [0.46, 3.50] |
| Opening time: 9am-8pm * | 13.57  [11.33, 15.80] | 13.83  [12.05, 15.61] | 13.39  [11.09, 15.69] | 13.83  [12.05, 15.61] |
| Opening time: 2pm-8pm * | 6.64  [4.80, 8.48] | 3.44  [2.00, 4.88] | 6.53  [4.43, 8.62] | 3.44  [2.00, 4.88] |
| Availability: Every weekday: Monday – Friday ** | -7.99  [-10.45, -5.54] | -2.46  [-3.69, -1.24] | -7.97  [-9.96, -5.97] | -2.46  [-3.69, -1.24] |
| Availability: 1 day every 2 months: Monday – Friday ** | -16.52  [-18.75, -14.29] | -18.31  [-19.79, -16.84] | -16.54  [-18.84, -14.24] | -18.31  [-19.79, -16.84] |
| Availability: 1 day every 2 months: Saturday or Sunday ** | -30.73  [-33.26, -28.19] | -33.65  [-35.46, -31.84] | -30.63  [-33.12, -28.14] | -33.65  [-35.46, -31.84] |
| Max donations: 5 *** | 7.44  [5.48, 9.39] | 11.81  [10.39, 13.23] | 7.45  [5.79, 9.11] | 11.81  [10.39, 13.23] |
| Max donations: 6 *** | 7.54  [5.64, 9.43] | 17.72  [16.44, 19.00] | 7.61  [5.83, 9.40] | 17.72  [16.44, 19.00] |

Reference category: *9am-12pm and 2pm-5pm, **Every day: Monday to Sunday, ***4 donations per year. **^+^** CI calculated with non-parametric bootstrap

**Table S10. MRS estimates (95% CI)^+^ for each categorical survey attribute versus travel time for SP-ordered categorical and DCE survey responses from 4,179 females for the full sample and after excluding opt-out respondents.**

|  | **Model 3:**  **MNL with random effects**  **(full sample)** | | **Model 4:**  **MNL with random effects**  **(excluding opt-out respondents)** | |
| --- | --- | --- | --- | --- |
|  | **SP-ordered**  **Categorical** | **DCE** | **SP-ordered**  **categorical** | **DCE** |
| Health report | 3.24  [1.96, 4.52] | 11.29  [10.31, 12.27] | 3.26  [1.98, 4.53] | 11.29  [10.31, 12.27] |
| Opening time: 9am-5pm * | 4.01  [2.40, 5.61] | 12.84  [11.79, 13.89] | 4.01  [2.07, 5.96] | 12.84  [11.79, 13.89] |
| Opening time: 9am-8pm * | 18.17  [16.01, 20.34] | 21.92  [20.73, 23.12] | 18.08  [16.11, 20.04] | 21.92  [20.73, 23.12] |
| Opening time: 2pm-8pm * | 11.01  [9.00, 13.03] | 8.46  [7.23, 9.68] | 10.95  [8.94, 12.96] | 8.46  [7.23, 9.68] |
| Availability: Every weekday: Monday – Friday ** | -9.75  [-12.07, -7.44] | -2.71  [-3.99, -1.43] | -9.76  [-11.92, -7.60] | -2.71  [-3.99, -1.43] |
| Availability: 1 day every 2 months: Monday – Friday ** | -19.75  [-22.07, -17.43] | -21.56  [-23.06, -20.07] | -19.81  [-22.13, -17.49] | -21.56  [-23.06, -20.07] |
| Availability: 1 day every 2 months: Saturday or Sunday ** | -26.38  [-28.92, -23.85] | -28.92  [-30.17, -27.66] | -26.38  [-28.71, -24.06] | -28.92  [-30.17, -27.66] |
| Max donations: 4 *** | 4.23  [2.73, 5.73] | 11.68  [10.91, 12.46] | 4.27  [3.07, 5.46] | 11.68  [10.91, 12.46] |

Reference category: *9am-12pm and 2pm-5pm, **Every day: Monday to Sunday, ***3 donations per year

**Table S11. Marginal rates of substitution from SP-ordered categorical and DCE survey responses from random effects multinomial logistic regression model (model 3) for males weighted by eligible NHSBT donor characteristics.**

|  | **Random effects**  **multinomial logit** | | **Random effects**  **multinomial logit**  **population weighted** | |
| --- | --- | --- | --- | --- |
|  | **SP-ordered**  **categorical** | **DCE** | **SP-ordered**  **categorical** | **DCE** |
| Health report | 2.67  [1.23, 4.10] | 8.49  [7.47, 9.50] | 3.49  [0.63, 6.35] | 8.74  [6.26, 11.22] |
| Opening time: 9am-5pm * | 2.34  [0.46, 4.21] | 1.98  [0.46, 3.50] | 3.36  [-1.09, 7.81] | 0.40  [-2.82, 3.63] |
| Opening time: 9am-8pm * | 13.57  [11.33, 15.80] | 13.83  [12.05, 15.61] | 17.85  [12.08, 23.61] | 18.59  [14.41, 22.76] |
| Opening time: 2pm-8pm * | 6.64  [4.80, 8.48] | 3.44  [2.00, 4.88] | 15.62  [10.34, 20.91] | 9.35  [6.07, 12.64] |
| Availability: Every weekday: Monday – Friday ** | -7.99  [-10.45, -5.54] | -2.46  [-3.69, -1.24] | -13.92  [-19.12, -8.72] | -7.86  [-11.37, -4.34] |
| Availability: 1 day every 2 months: Monday – Friday ** | -16.52  [-18.75, -14.29] | -18.31  [-19.79, -16.84] | -19.80  [-24.90, -14.70] | -25.41  [-29.51, -21.30] |
| Availability: 1 day every 2 months: Saturday or Sunday ** | -30.73  [-33.26, -28.19] | -33.65  [-35.46, -31.84] | -27.63  [-32.51, -22.74] | -33.40  [-37.95, -28.84] |
| Max donations: 5 *** | 7.44  [5.48, 9.39] | 11.81  [10.39, 13.23] | 9.94  [5.65, 14.23] | 11.44  [8.37, 14.52] |
| Max donations: 6 *** | 7.54  [5.64, 9.43] | 17.72  [16.44, 19.00] | 9.80  [5.63, 13.96] | 12.72  [9.93, 15.50] |

Reference category: *9am-12pm and 2pm-5pm, **Every day: Monday to Sunday, ***4 donations per year

**Table S12. Marginal rates of substitution from SP-ordered categorical and DCE survey responses from random effects multinomial logistic regression model (model 3) for females weighted by eligible NHSBT donor characteristics**

|  | **Random effects**  **multinomial logit** | | **Random effects**  **multinomial logit**  **population weighted** | |
| --- | --- | --- | --- | --- |
|  | **SP-ordered**  **categorical** | **DCE** | **SP-ordered**  **categorical** | **DCE** |
| Health report | 3.24  [1.96, 4.52] | 11.29  [10.31, 12.27] | 3.53  [1.60, 5.47] | 9.44  [7.94, 10.94] |
| Opening time: 9am-5pm * | 4.01  [2.40, 5.61] | 12.84 [11.79, 13.89] | 4.01  [1.43, 6.59] | 12.33  [10.68, 13.99] |
| Opening time: 9am-8pm * | 18.17  [16.01, 20.34] | 21.92  [20.73, 23.12] | 24.97  [21.17, 28.78] | 25.06  [22.77, 27.36] |
| Opening time: 2pm-8pm * | 11.01  [9.00, 13.03] | 8.46  [7.23, 9.68] | 16.59  [13.26, 19.92] | 13.92  [12.00, 15.83] |
| Availability: Every weekday: Monday – Friday ** | -9.75  [-12.07, -7.44] | -2.71  [-3.99, -1.43] | -12.70  [-16.17, -9.24] | -8.25  [-10.04, -6.46] |
| Availability: 1 day every 2 months: Monday – Friday ** | -19.75  [-22.07, -17.43] | -21.56  [-23.06, -20.07] | -24.22  [-27.79, -20.64] | -24.77  [-26.82, -22.72] |
| Availability: 1 day every 2 months: Saturday or Sunday ** | -26.38  [-28.92, -23.85] | -28.92  [-30.17, -27.66] | -26.98  [-30.75, -23.22] | -26.97  [-28.88, -25.06] |
| Max donations: 4 *** | 4.23  [2.73, 5.73] | 11.68  [10.91, 12.46] | 4.94  [3.08, 6.80] | 8.91  [7.76, 10.06] |

Reference category: *9am-12pm and 2pm-5pm, **Every day: Monday to Sunday, ***3 donations per year

**Figure S1. Trace of bootstrap results for SP-ordered categorical survey responses from random effects multinomial logistic regression model (model 3) for males**


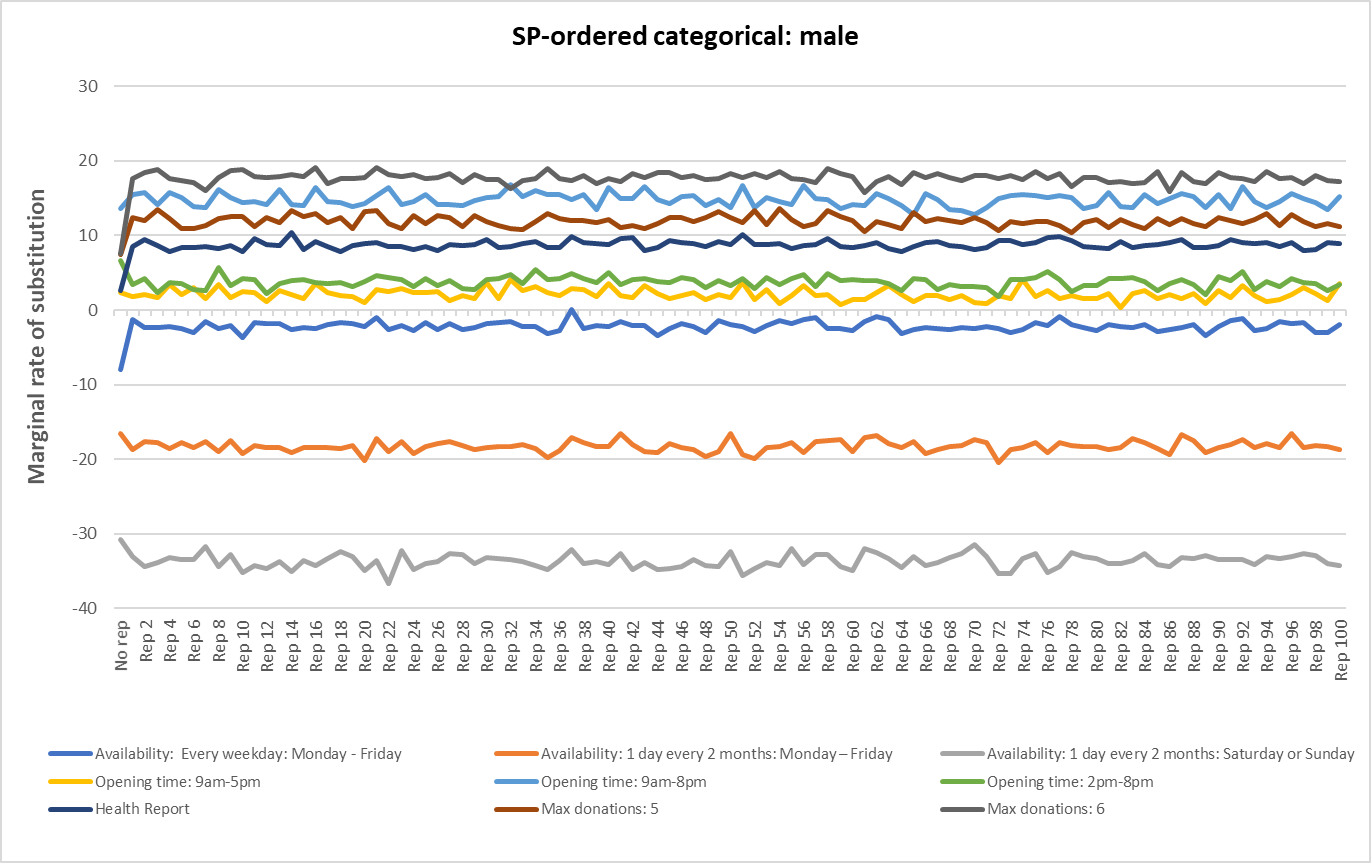


**Figure S2. Trace of bootstrap results for DCE survey responses from random effects multinomial logistic regression model (model 3) for males**


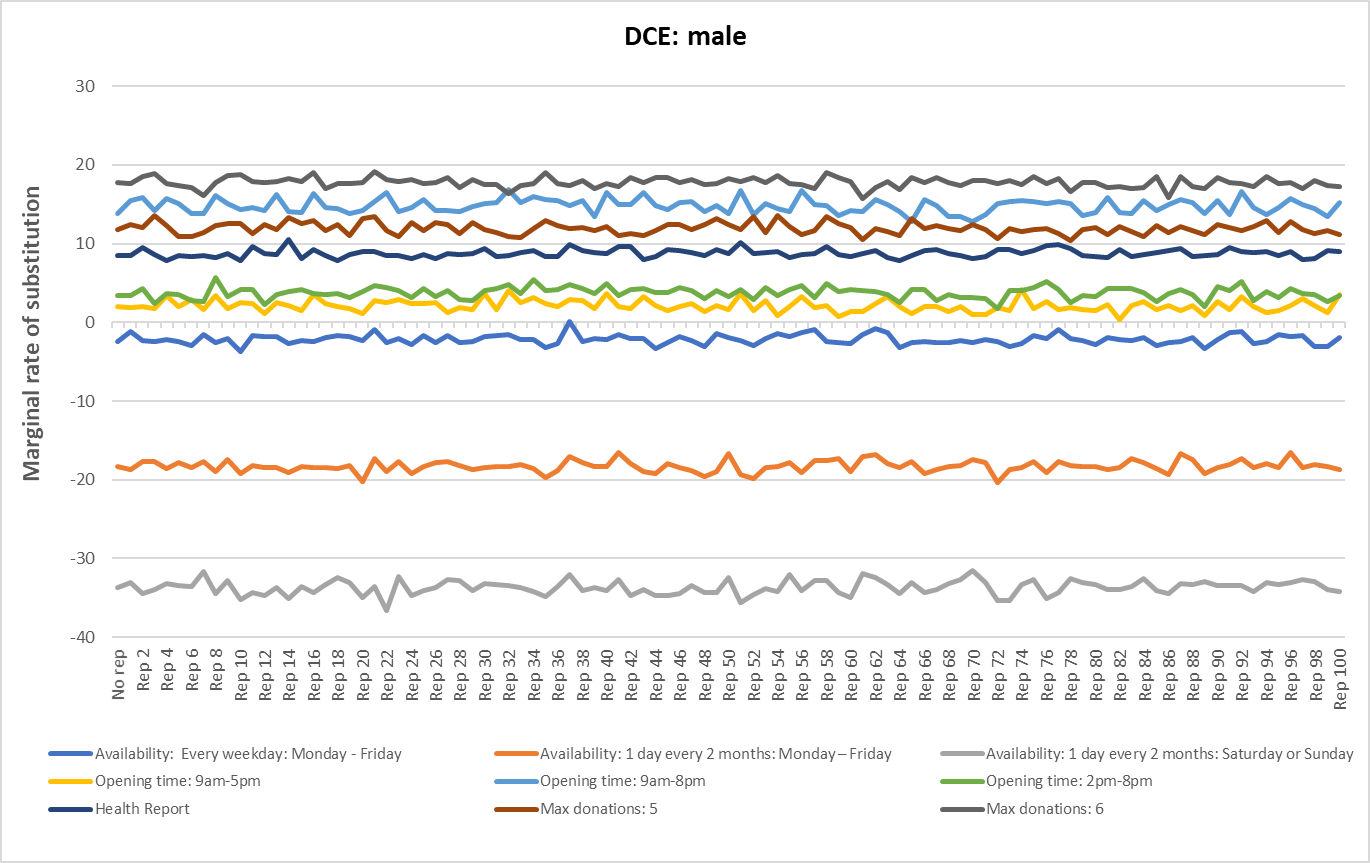


**Figure S3. Trace of bootstrap results for SP-ordered categorical survey responses from random effects multinomial logistic regression model (model 3) for females**


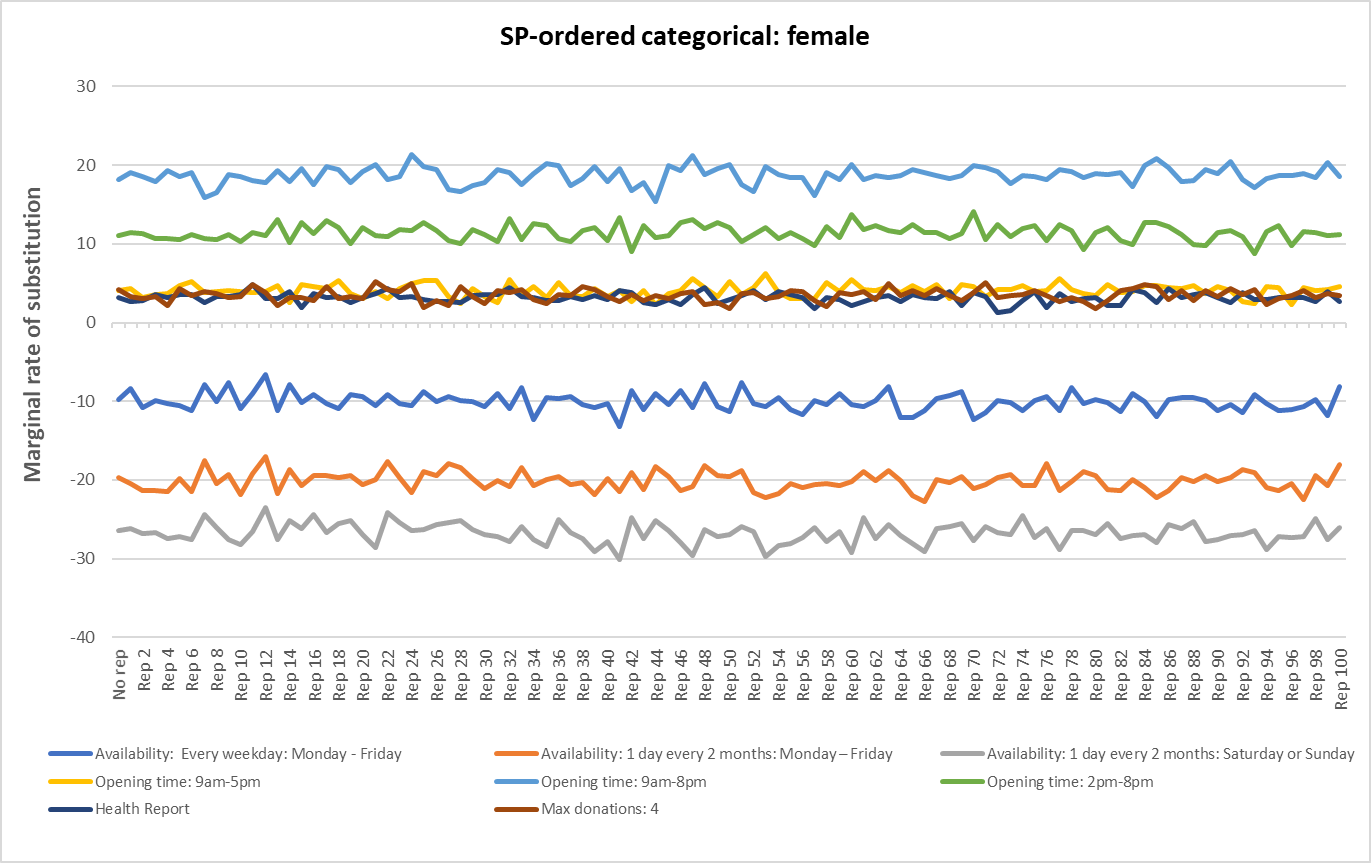


**Figure S3. Trace of bootstrap results for SP-ordered categorical and DCE survey responses from random effects multinomial logistic regression model (model 3) for females**

**
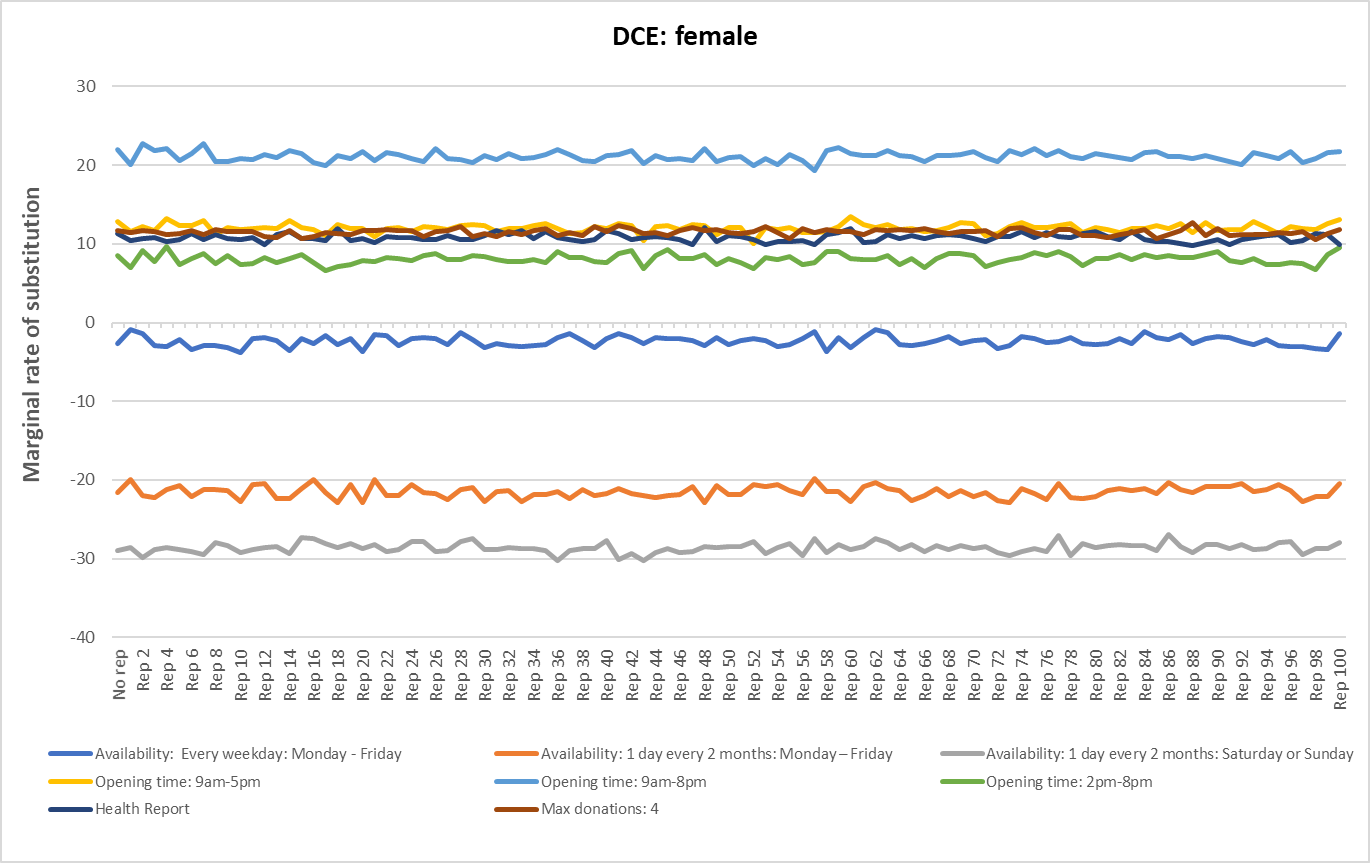
**
