# Supplementary material for: A Comparison of Ordered Categorical versus Discrete Choices within a Stated Preference Survey of Whole-Blood Donors
Source: Med Decis Making. 2022 Dec 24;43(3):362–73. doi: 10.1177/0272989X221145048 (PMC10021117; doi:10.1177/0272989X221145048)
Supplement: sj-docx-2-mdm-10.1177_0272989X221145048 – Supplemental material for A Comparison of Ordered Categorical versus Discrete Choices within a Stated Preference Survey of Whole-Blood Donors [file sj-docx-2-mdm-10.1177_0272989X221145048.docx]

**Theoretical foundations of stated preference (SP) approaches**

SP approaches for eliciting individual preferences require that respondents are presented with a series of alternatives, and are asked to state their relative preferences. The theoretical foundations of the most common form of SP design, the Discrete Choice Experiment (DCE), are Lancaster’s consumer theory (Lancaster, 1966) and Random Utility Theory (RUT) (McFadden, 1974). Here an individual chooses the alternative that provides the greatest utility, where the utility of each alternative depends on an observed (deterministic) component comprised of attributes, levels and an unobserved random error. An individual *i*’s total utility U from an alternative *j* is

$$U_{ij}=\sum_{k=1}^{K} \beta_{k}x_{kij}+\epsilon_{ij}$$

reflecting a linear combination of attributes $x_{kij}$ weighted by their importance $\beta_{k}$, and a random utility component $\epsilon_{ij}$.

Most commonly used DCE framing requires individual *i* to make a binary choice about whether or not to choose alternative *j* from the competing alternatives within the choice set of 1, …, *J* , with the probability of choosing a particular alternative given by:

$${Pr}_{ij}=\frac{exp (\sum_{k=1}^{K} \beta_{k}x_{kij})}{\sum_{h=1}^{J} exp (\sum_{k=1}^{K} \beta_{k}x_{kih})}$$

The model specification commonly applied to then estimate the probability of a particular, binary choice, is the logit model.

When the SP survey is required to provide information for a cost-effectiveness analysis (CEA), the choice task can be formulated to elicit preferences about the individual’s *level* of engagement, or frequency of participation (*SP- ordered categorical*). For example, in the context of blood donation, it is important not only to understand what motivates whether or not donors choose to donate, but also their *frequency* of donation. This richer preference information can be obtained by framing DCE choice tasks to allow respondents to express their level of engagement according to the alternative choices. The initial HEMO study SP design (*SP-ordered categorical*) required responders to express relative preferences for alterative choices according to donation frequency defined as an ordered categorical response variable. An individual donor was presented with a series of choice alternatives and asked to state their preferred level of a categorical outcome (donation frequency) for each choice alternative. Similar to a DCE, the theoretical foundations for this choice mechanism are utility maximisation and RUT. Consider a utility function denoted by

$$U=U(x,q,\beta,\varepsilon)$$

where *x* is a vector of donation level, *q* is a matrix of attributes and levels attached to choose alternatives, $\beta$ and $\varepsilon$ are vectors of parameters and unobserved error terms respectively. Given the opportunity cost, *c*, of choice alternatives and the overall time constraint, *y*, the utility maximisation problem is

$$\max_{x} U\left( x,q,\beta,\varepsilon\right) such that c\leq y,x\geq0$$

A donor expresses their level of preference for the alternative donation choices, given the attributes and their levels, and implicitly chooses the category of donation frequency to maximise utility. This choice will be guided by the relative marginal utility of alternative donation frequencies ($x_{i}, i=1,\ldots.n$), and by their relative opportunity cost, according to the level of constraint they imply for the donor.

$$\frac{\partial U}{\partial x}=\frac{\partial c}{\partial x}$$

With the *SP-ordered categorical* approach, individuals were presented with a set of options described by attribute levels a, b, c and asked to choose their preferred donation frequency.

| Attribute a | a^+^ | a^+^ | a^+^ | a^+^ | a^+^ |
| --- | --- | --- | --- | --- | --- |
| Attribute b | b^+^ | b^+^ | b^+^ | b^+^ | b^+^ |
| Attribute c | c^+^ | c^+^ | c^+^ | c^+^ | c^+^ |
| Preferred donation option (frequency) | zero | one | two | three | four |

Under the DCE approach respondents were presented with two options, and asked which they preferred.

| Attribute a | a* | a’ |
| --- | --- | --- |
| Attribute b | b* | b’ |
| Attribute c | c* | c’ |
| Preferred donation option | Option 1 | Option 2 |

The theoretical underpinning of RUT is equally applicable to each of the SP approaches as described above. However, the differences between SP-ordered categorical and DCE approach are:

(i) SP-ordered categorical has one more dimension (donation frequency) and

(ii) The SP-ordered categorical approach requires the individual chooses one from a set of five, whereas with DCE the individual chooses one from a set of two.

Given a common set of attributes and levels, and invoking the general principle that respondents will maximise utility, we expect individuals to draw on the same set of preferences, whether SP tasks and questions are designed to provide a ‘binary’ or ‘ordered categorical’ response. However, the alternative definitions of the response variable imply that the choice questions (tasks) will be formulated in different ways. We examine whether these alternative ways of eliciting these same sets of preferences, provide different estimates of the MRSs.
