# Supplementary material for: A Comparison of Ordered Categorical versus Discrete Choices within a Stated Preference Survey of Whole-Blood Donors
Source: Med Decis Making. 2022 Dec 24;43(3):362–73. doi: 10.1177/0272989X221145048 (PMC10021117; doi:10.1177/0272989X221145048)
Supplement: sj-docx-3-mdm-10.1177_0272989X221145048 – Supplemental material for A Comparison of Ordered Categorical versus Discrete Choices within a Stated Preference Survey of Whole-Blood Donors [file sj-docx-3-mdm-10.1177_0272989X221145048.docx]

**Latent Class Analysis of HEMO Blood Donation Preference Data**

We conducted a latent class analysis (LCA) using LatentGOLD 6.0 following established guidelines for model selection. to eight-class models were built with and without scale adjustment. Scale adjustment was limited to two scales as models with more than two scales failed to converge. Model selection first considered BIC and then considered class separation, size and homogeneity. As male and female participants completed different choice tasks, their data were analyzed separately. For selected models, cross-tabulations of class and demographic characteristics and donor behaviour were reviewed to identify potential predictors of class membership.

*Males*

A 6-class model without a scale parameter was preferred. The largest class comprised 25% of the sample and the smallest class comprised 12% of the sample. In general, classes preferred: to complete the health report; regular daily availability, and shorter travel distances. No classes had a preference for one day a month availability on Saturday or Sunday. However, some classes had strong preferences for evening and afternoon availability (e.g., Class 1) and some had preferences against only evening or afternoon availability (e.g., Class 3). All classes, except Class 1, preferred donating 6 times per year to donating 5 or 3 times per year, however the strength of the preference varied across groups. Review of cross tabulations did not find strong associations between classes and demographic characteristics or donor behaviour for all classes.

*Females*

A 5-class model without a scale parameter was preferred. The largest class comprised 27% of the sample and the smallest class comprised 14% of the sample. In general, like with male participants, classes preferred: to complete the health report; regular daily availability; and shorter travel times. No classes had a preference for one day a month availability on Saturday or Sunday. Notably, only Class 5, the smallest class, preferred weekday availability (either regular weekday availability or one day per month weekday availability). Similarly, all classes except for Class 5 preferred donating 4 times per year to donating 3 times per year. Review of cross tabulations did not find strong associations between classes and demographic characteristics or donor behaviour for all classes.

**Table 1. Parameter Estimates for 6-Class Model for male participants**

| **Attributes** | **Class 1 (24%)** |  | **Class 2 (18%)** | **Class 3 (17%)** | **Class 4 (15%)** | **Class 5 (15%)** | **Class 6 (12%)** | **Wald** | **p-value** |
| --- | --- | --- | --- | --- | --- | --- | --- | --- | --- |
| **Health Report** |  |  |  |  |  |  |  |  |  |
| No | 0 |  | 0 | 0 | 0 | 0 | 0 | 84.2436 | 4.70E-16 |
| Yes | 0.1373 |  | 0.3278 | 2.35 | 7.8765 | 0.5664 | -0.7715 |  |  |
| **Opening Hours** |  |  |  |  |  |  |  |  |  |
| 1 - 9 to 12 & 2 to 5 | 0 |  | 0 | 0 | 0 | 0 | 0 | 521.6736 | 2.90E-99 |
| 2 - 9 to 5 | -0.0644 |  | 0.6108 | -0.3819 | -12.2368 | 9.3915 | -0.5748 |  |  |
| 3 - 9 to 8 | 2.0427 |  | 0.5553 | -1.058 | -5.1992 | 9.5937 | -1.8718 |  |  |
| 4 - 2 to 8 | 1.3471 |  | -0.4427 | -4.5935 | -0.6017 | -0.6414 | -0.1732 |  |  |
| **Operating Days** |  |  |  |  |  |  |  |  |  |
| Every day | 0 |  | 0 | 0 | 0 | 0 | 0 | 532.1659 | 1.80E-101 |
| Every weekday | -0.7305 |  | 0.1238 | -0.3615 | -4.8028 | 2.0827 | -0.1669 |  |  |
| One day a month Mon-Fri | -1.4286 |  | 0.1341 | -1.1905 | -12.8512 | 0.531 | -1.0016 |  |  |
| One day a month Sa-Su | -0.5762 |  | -3.1617 | -1.4708 | -21.2593 | -14.6859 | -1.5888 |  |  |
| **Maximum Donation** |  |  |  |  |  |  |  |  |  |
| 3 donations | 0 |  | 0 | 0 | 0 | 0 | 0 | 110.2594 | 5.30E-18 |
| 5 donations | -0.1006 |  | -0.0315 | 2.1560 | 4.9109 | -0.5395 | 3.5370 |  |  |
| 6 donations | -0.0046 |  | 0.1355 | 2.6067 | 10.8919 | 0.3964 | 7.3156 |  |  |
| **Travel Time** |  |  |  |  |  |  |  |  |  |
| Increasing (continuous) | -0.0338 |  | -0.0264 | -0.1502 | -0.4012 | -0.9923 | -0.0206 | 279.8884 | 1.70E-57 |

**Table 2: Parameter Estimates for 5-Class Model for female participants**

| **Attributes** | **Class 1 (27%)** | **Class 2 (22%)** | **Class 3 (18%)** | **Class 4 (18%)** | **Class 5 (14%)** | **Wald** | **p-value** |
| --- | --- | --- | --- | --- | --- | --- | --- |
| **Health Report** |  |  |  |  |  |  |  |
| No | 0 | 0 | 0 | 0 | 0 | 126.5584 | 1.30E-25 |
| Yes | 1.2746 | -0.2427 | 5.3166 | 0.9134 | 3.0299 |  |  |
| **Opening Hours** |  |  |  |  |  |  |  |
| 1 - 9 to 12 & 2 to 5 | 0 | 0 | 0 | 0 | 0 | 761.803 | 1.20E-152 |
| 2 - 9 to 5 | 0.996 | 0.1767 | 1.7315 | -0.2969 | 9.1295 |  |  |
| 3 - 9 to 8 | 1.2617 | 2.7619 | 3.1368 | -1.4125 | 11.3503 |  |  |
| 4 - 2 to 8 | -0.1634 | 1.9614 | 3.0843 | -4.0304 | 8.0107 |  |  |
| **Operating Days** |  |  |  |  |  |  |  |
| Every day | 0 | 0 | 0 | 0 | 0 | 891.0137 | 3.00E-180 |
| Every weekday | -0.4045 | -1.9508 | -2.3432 | -0.0261 | 13.6918 |  |  |
| One day a month Mon-Fri | -1.6056 | -3.1001 | -2.1888 | -1.841 | 6.4045 |  |  |
| One day a month Sa-Su | -2.0606 | -1.7078 | -1.6401 | -2.9179 | -3.5014 |  |  |
| **Maximum Donation** |  |  |  |  |  |  |  |
| 3 donations | 0 | 0 | 0 | 0 | 0 | 190.5651 | 3.00E-39 |
| 4 donations | 0.732 | 0.9192 | 0.659 | 2.0561 | -1.2607 |  |  |
| **Travel Time** |  |  |  |  |  |  |  |
| Increasing (continuous) | -0.0305 | -0.0561 | -0.2798 | -0.152 | -0.2579 | 263.5612 | 6.80E-55 |
